# Comparison of Fetal Cerebellum and Cisterna Magna Length by 2D and 3D Ultrasonography between 18 and 24 Weeks of Pregnancy

**DOI:** 10.5402/2012/286141

**Published:** 2012-11-14

**Authors:** Fernanda Silveira Bello de Barros, Luiz Cláudio de Silva Bussamra, Edward Araujo Júnior, Leonardo da Silva Valladão de Freitas, Luciano Marcondes Machado Nardozza, Antonio Fernandes Moron, José Mendes Aldrighi

**Affiliations:** ^1^Department of Obstetrics and Gynecology, Medical Science College of Santa Casa of São Paulo (FCMSCSP), 05303-000 São Paulo, SP, Brazil; ^2^Department of Obstetrics, Federal University of São Paulo (UNIFESP), 05303-000 São Paulo, SP, Brazil

## Abstract

To compare the fetal cerebellum and cisterna magna length measurements by means of two- (2DUS) and three-dimensional (3DUS) ultrasonography using the three-dimensional extended imaging (3D XI), a cross-sectional study with 69 healthy pregnant women between 18 and 24 weeks was performed. For the measurements by 2DUS, the axial planes were used and for the 3DUS a sequence of adjacent axial slices (multislice view). To evaluate the difference between the two techniques, we used the Wilcoxon test. To evaluate the correlation between the cerebellum and cisterna magna length measurements and the gestational age, we used the Spearman correlation coefficient (*r*). For the calculation of reproducibility, we used the intraclass correlation coefficient (ICC). The mean of the transverse and anterior-posterior diameter of cerebellum and cisterna magna by 3DUS was 9.23 and 6.62 mm, respectively. It was observed that the average of the measurements obtained by 3DUS was significantly higher, 0.76 and 1.02 mm for the length of the cerebellum and cisterna magna, respectively (*P* < 0.001). There was a high correlation between the length measurement of the cerebellum 3D (*r* = 0.940, *P* < 0.001), but low correlation of cisterna magna 3D (*r* = 0.462, *P* = 0.080) with the gestational age. There was good intra- and interobserver reproducibility for the cerebellum and cisterna magna 3D with ICC = 0.792 , 0.668, 0.691, and 0.287, respectively. The measurements of the fetal cerebellum and cisterna magna length by 3DUS using the software 3D XI were significantly higher than those obtained by 2DUS.

## 1. Introduction

The embryonic development of the cerebellum derives from the hindbrain from the fifth week, originating from the thickening of the dorsal alar plates. Anatomically it is formed by a median portion, the vermis, connected to two large lateral masses, the cerebellar hemispheres. The cisterna magna is located in the posterior fossa and occupies the space between the underside of the cerebellum, the posterior side of the bulb and the roof of the fourth ventricle. It continues caudally with the spinal subarachnoid space and binds to the fourth ventricle through the opening median [1].

At ultrasonography the cerebellar hemispheres are usually low to moderately echogenic, connected above by an echogenic cerebellar tentorium. The vermis is a highly echogenic structure, which can be recognized as an oval density in the midline of the axial and sagittal plans [2]. The cerebellum and cisterna magna can be observed on ultrasound around 11 weeks, but the formation of the cerebellar vermis ends around 18 weeks. For this reason, we must be careful in early diagnosis of the posterior fossa anomalies before this period, due to the possibility of a pathological image simulation [3, 4].

The measurement of the transverse cerebellar diameter by two-dimensional ultrasonography (2DUS) is a very reliable parameter in the evaluation and early detection of intrauterine growth restriction [5]. On the other hand, an adequate evaluation of the posterior fossa abnormalities allows the diagnosis of the cisterna magna as the Dandy-Walker complex, megacisterna magna, and Blake's cyst.

The three-dimensional ultrasonography (3DUS) appeared as an important tool in the evaluation of fetal central nervous system in the late 1990s, because of the development of high resolution endocavitary volumetric transducers [6]. Currently, with the development of new software like volume contrast imaging allows adequate evaluation of posterior fossa anomalies as well as the biometry of the cerebellar vermis [7, 8].

The three-dimensional extended imaging software (3D XI-Medison, Seoul, Korea) is composed of three programs: multislice view, volume CT view, and oblique view. The multislice consists of multiple sequential and adjacent planes arranged in the set screen (sagittal, axial, and coronal). The reference plane, number of images arranged in the screen (1 × 1, 2 × 1, 3 × 2, 4 × 3, or 6 × 2), orientation and rotation of the image, the magnitude of the magnification, and depth and range between the planes (0.5 to 5.0 mm) can be adjusted according to the region of interest [9]. A preliminary study evaluated the application of the multislice view in fetal central nervous system anomalies, showing potential benefits [10]. Up to date there are no studies evaluating the measures of the fetal cerebellum and cisterna magna by 3DUS using the multislice view.

The objective of this study is to compare the measurements of transverse cerebellar and anterior-posterior cisterna magna diameter obtained by 2D and 3DUS using the software 3D XI in normal pregnant women between 18 and 24 weeks.

## 2. Methods

A prospective cross-sectional study between March 2010 and February 2011, involving 69 normal pregnant women between 18 and 24 weeks was performed. This study was approved by the Research Ethics Committee of the Irmandade da Santa Casa de Misericórdia de São Paulo, Brazil, and the patients who agreed to participate signed a term of consent.

This study was carried out at the Department of Obstetrics and Gynecology, Faculty of Medical Sciences of Santa Casa of São Paulo (FCMSCSP). The patients were randomly selected, and all evaluation made by a single examiner (FSBB), with five years experience in obstetric ultrasound. The examinations were performed on a SonoAce X8 (Samsung-Medison, Seoul, Korea) device equipped with multifrequency volumetric convex transducer (3–7 MHz). The criteria for inclusion were (1) unique pregnancy with live fetus and (2) gestational age evaluated by last menstrual period and confirmed by ultrasound performed until the 14th week (crown-rump length-CRL: 4–84 mm). Exclusion criteria were (1) pregnant women carrying fetuses with structural anomalies detected at the time of the examination and (2) pregnant women carrying chronic diseases that would interfere with fetal growth.

Initially, a realtime 2D evaluation was performed in order to evaluate the biometry, morphology, and quantification of amniotic fluid volume. For the 2D measurement of transverse cerebellar and anterior-posterior cisterna magna diameter, it was performed a modified transversal slice of the fetal head slightly angled, through the thalamus, cerebellar hemispheres, cisterna magna, cave of septum pellucidum, the occipital bone, and nuchal fold. 

An insonation angle of the occipital bone was chosen, taking care that it was focused on an angle of 30°. It was performed a single measurement of transverse cerebellar and antero-posterior of the fetal cisterna magna diameter in each mother, and this image is saved in the memory of the device.

**Figure 1:**
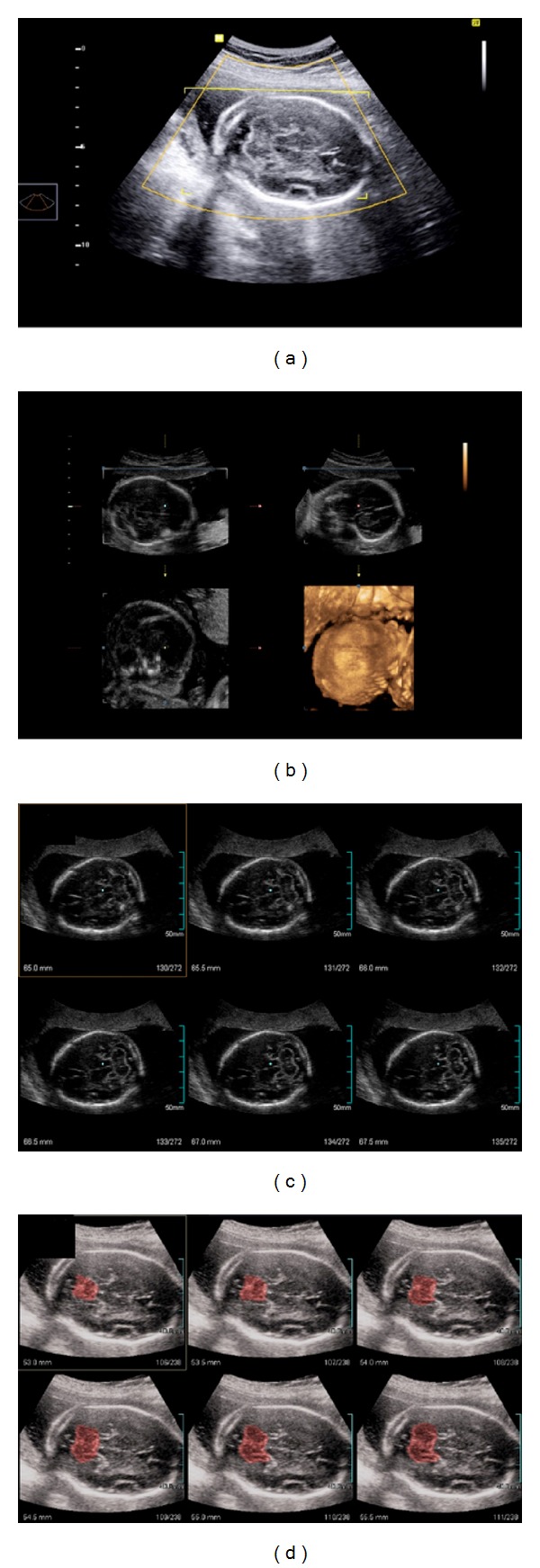
(a) Three-dimensional scan at the pattern plan level of measurement for the transverse cerebellar and anterior-posterior cisterna magna diameter (thalamus, cerebellar hemispheres, cisterna magna, cavum septum pellucidum, the occipital bone, and nuchal fold) encompassing the entire fetal skull (ROI-region of interest). (b) Multiplanar mode: axial (top right), sagittal (lower right), and coronal (top left). (c) Multislice view: 2 × 3 arrangement (two rows and three columns), with slice thickness cut of 0.5 mm. (d) Adjacent sequential images, showing plan above the cerebellum (in red).

The three-dimensional volume acquisition was performed on the same 2D plane in which was performed the measurements of the transverse cerebellar diameter and anterior-posterior cisterna magna, to encompass the entire fetal skull (ROI-region of interest) (Figure 1(a)). In order to standardize all 3D measurements, the following preset was used on the device: scanning—3D static; display mode—three-dimensional extended imaging (multislice view); scanning speed—slow; angle scanning—70°; overall gain of the device—50%. After the three-dimensional scanning, the image was displayed in the multiplanar mode (axial, sagittal, and coronal) (Figure 1(b)). The volumes were saved in the device memory and then stored on compact discs (CDs) and transferred to a personal computer. The analyses were performed offline in the same apparatus in a time period of 30 to 120 days after the volumetric capture.

For measurements of the transverse cerebellar and anterior-posterior cisterna magna diameters the program multislice view on 3 × 2 (two rows and three columns) was chosen, with slice thickness of 0.5 mm (Figure 1(c)). The buttons 3 and 4 of the device were maneuvered, for navigation between tomographic slices, until in one of them the image of the cerebellum disappeared in higher plan than that of the posterior fossa (Figure 1(d)). From this point, the 1 × 1 (one row and one column) option was selected and the number of the image was recorded. With the presence of only one tomographic slice on the screen, the button 4 of the apparatus was rotated clockwise or counterclockwise, depending on the fetus position, leading the exposition of a lower plan, successively until the outline of the cerebellum began to appear. Following, each plan of the cerebellum and the cisterna magna was measured, with a difference of 0.5 mm between them to the normal cerebellum outline disappeared and the bone of the skull base could be observed. On average, measurements of the transverse cerebellar diameter and the antero-posterior diameter of the cisterna magna were performed in 25 consecutive plans in each volume, being considered as the value of the measurement of the cerebellum and the cisterna magna by 3D XI method the highest value obtained. The average time for manipulation of the 3D volume measurement of the cerebellum and the cisterna magna was 180 seconds.

The data were transferred to an Excel 2007 (Microsoft Corp, Redmond, WA, USA) spreadsheet and analyzed by the Statistical Package for Social Sciences (SPSS Inc., Chicago, IL, USA) version 19.0 for Windows. For the 2D and 3D measurements of the transverse cerebellar diameter and antero-posterior cisterna magna, mean, median, maximum, and minimum values were calculated. And for the 3D measurements percentiles 5, 10, 90, and 95 were also calculated in each gestational age evaluated. To evaluate the difference between the two techniques the Wilcoxon test was used. To evaluate the correlation of 2D and 3D, measurements of the transverse cerebellar diameter and antero-posterior cisterna magna as well as measurements of biparietal diameter (BPD) and head circumference (HC) according to the gestational age, the Spearman's correlation coefficient (*r*) was used. In cases of high correlation, polynomial regression models, with adjustments by the coefficient of determination (*R* ^2^) were performed. To assess intraobserver reproducibility, the first examiner (FSBB) held a second 2D and 3D measures in 8 cases randomly selected 7 days after the first. For interobserver reproducibility, a second examiner (LSVF), with the same experience in obstetric ultrasound performed a third measure of these same 8 cases, and the results were armored one of the other. For this purpose, to calculate the reproducibility intraclass correlation coefficient (ICC) and Bland-Altman plots were used. It is considered poor correlation ICC < 0.40; satisfactory ICCs between 0.40 and 0.75 and excellent ICC ≥ 0.75 [11]. The Bland-Altman plots evaluate the average of measurements performed by one or two examiners plotted against the difference of their mean values with standard deviation 1.96 (limits of agreement) [12]. In all analysis we used a significance level (*P*) < 0.05.

## 3. Results

The 69 patients initially selected met the criteria for inclusion and exclusion, being allocated in the final statistical analysis. The age of the pregnant women ranged from 17 to 41 years, with an average of 26.93 years (standard deviation 6.10 years). The number of pregnancies ranged from 1 to 6, with an average of 2.19 pregnancies (standard deviation of 1.32 pregnancies).

**Figure 2:**
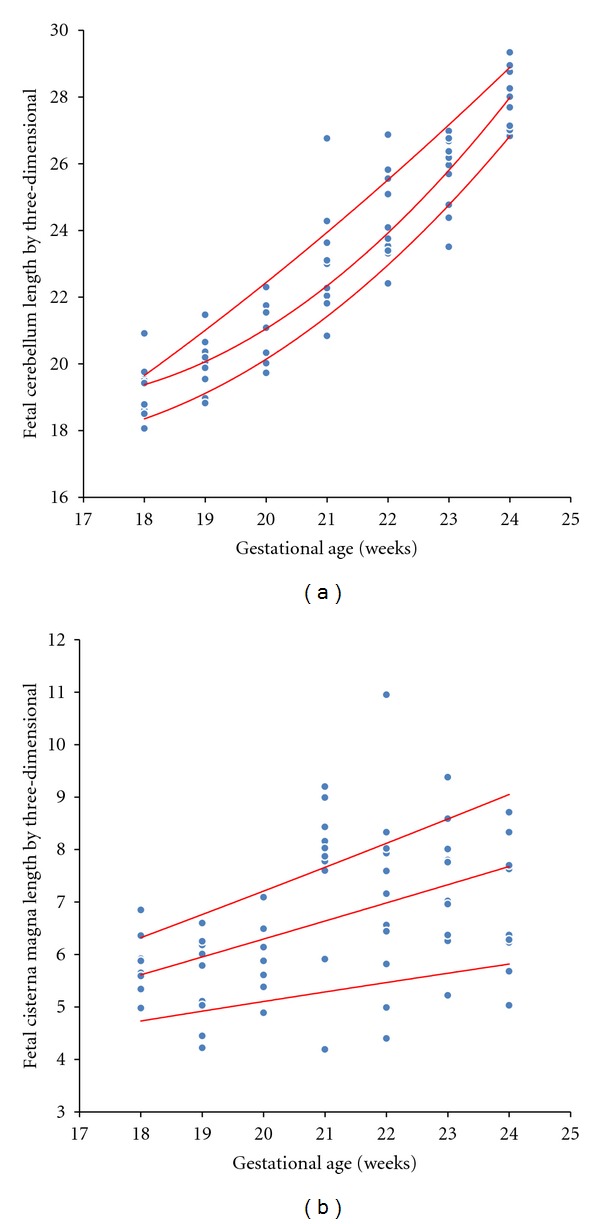
Scatter plot of correlation between the transverse cerebellar diameter (a) and antero-posterior diameter of the cisterna magna (b) by three-dimensional ultrasonography according to gestational age.

The average transverse and anterior-posterior cerebellum and cisterna magna diameter by 3DUS ranged from 9.23 ± 3.16 mm (18.06–29.34) and 6.62 ± 1.41 mm (4.19–10.45), respectively. The average transverse diameter and antero-posterior cerebellum and cisterna magna by 2DUS ranged from 22.33 ± 3.16 mm (18.14–29.10) and 5.60 ± 1.33 mm (3.18–9.32), respectively. It was observed that on average the measurements obtained by 3DUS were significantly higher, 0.76 and 1.02 mm for the length of the cerebellum and cisterna magna, respectively (*P* < 0.001) (Tables 1 and 2). There was a high correlation between the measurement of transverse cerebellar diameter by 3DUS with gestational age (*r* = 0.940, *P* < 0.001) as well as measures of DBP (*r* = 0.927, *P* < 0.001) and HC (*r* = 0.938, *P* < 0.001). The equation that best represented the correlation between the extent of the transverse cerebellar diameter and gestational age was of a second degree: DTC = 7.231 + 1.851 × GA − 0.027 × GA2 (*R* ^2^ = 0879). For the measurement of antero-posterior diameter of the cisterna magna, a low correlation between gestational age, BPD, and HC (*r* = 0.462, *P* < 0.001; *r* = 0.430, *P* < 0,001; *r* = 0.517, *P* < 0.001, resp.) (Figure 2) was observed. The low correlation between the antero-posterior diameter of the cisterna magna and gestational age did not allow the construction of polynomial regression models.

Tables 3 and 4 show the percentiles 5, 10, 90, and 95 for measurements of the transverse and anterior-posterior cerebellum and cisterna magna diameter at each gestational age evaluated.

**Figure 3:**
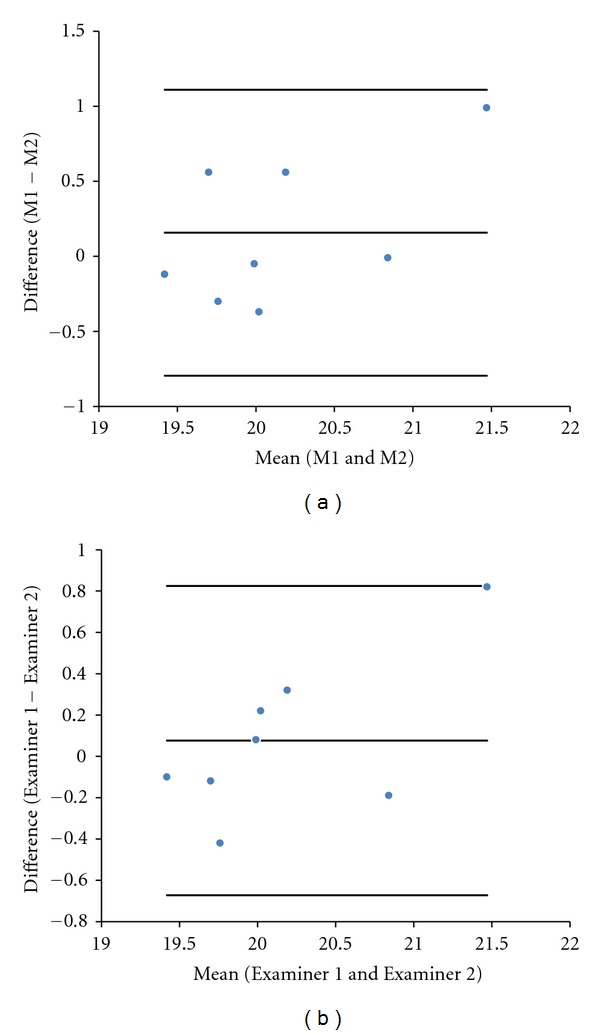
(a) Difference between the fetal cerebellum length measurement (M1 and M2) performed by a single examiner and plotted against the difference of their mean values. (b) Difference between the fetal cerebellum length measurement performed by two examiners (Examiner 1 and Examiner 2) plotted against the difference of their mean.

**Figure 4:**
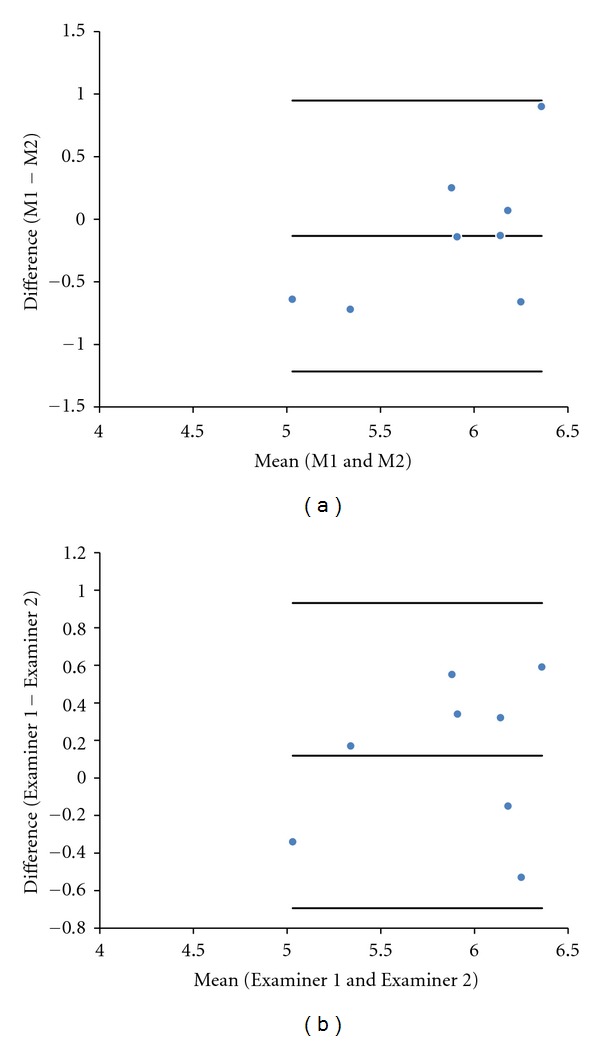
(a) Difference between the fetal cisterna magna length measurement (M1 and M2) performed by a single examiner and plotted against the difference of their mean values. (b) Difference between the fetal cisterna magna length measurement performed by two examiners (Examiner 1 and Examiner 2) plotted against the difference of their mean.

An ICC > 0.66 for all intra- and interobserver measurements of the cerebellum and cisterna magna length by 3DUS was observed, with the exception of the intraobserver measurement of the length of cisterna magna (ICC = 0.792, 0.668, 0.691, and 0.287, resp.). The mean differences as well the limits of agreement for intraobserver and interobserver reproducibility of the cerebellum and cisterna magna length by 3DUS were 0.16 mm (limits of agreement, −0.79; 1.11), −0.13 mm (limits of agreement, −1.22; 0.95), 0.08 mm (limits of agreement, −0.67; 0.82), and 0.12 mm (limits of agreement, −0.69; 0.93), respectively (Figures 3 and 4).

## 4. Discussion

The posterior fossa cystic malformations include abnormalities of the meninges (arachnoid cyst, megacisterna magna) and cerebellum (Dandy-Walker malformation and variants). The prenatal diagnosis of these malformations is of great importance for an adequate followup of pregnancy as well as the relatives counseling. The postnatal results are in general very bad in the Dandy-Walker malformation and variants, mainly as result of associated anomalies. The postnatal result of megacisterna magna is better, especially if isolated [13].

In this study, we compared the measurements of the transverse and antero-posterior cerebellum and cisterna magna diameters by 2D and 3DUS between 18 and 24 weeks. This interval was defined by the fact that the transverse cerebellar diameter showed the highest correlation with gestational age, its measurement in millimeters is similar to the gestational age in weeks [14]. Furthermore, the diagnosis of agenesis of the vermis, especially the partial cannot be performed before 18 weeks [15]. In relation to the antero-posterior diameter of the cisterna magna, in general, in this period the ultrasound of the second trimester for malformations evaluation will be performed, its normal measure being less than 10 mm.

In this study we observed that the transverse cerebellar and anterior-posterior cisterna magna diameters by 2D and 3DUS increased from 18 to 24 weeks, however, the cisterna magna measurements by 3DUS showed low correlation with the gestational age. In a cross-sectional study carried out by Vinkesteijn et al. [16] with 360 normal pregnant women between 17 and 34 weeks, the average of fetal transverse cerebellar diameter by 2DUS was 22.1 mm from 18 to 24 weeks, very similar to our results obtained using 2DUS (22.33 mm). In another cross-sectional study carried out by Koktener et al. [17] with 194 normal pregnant women between 16 and 24 weeks, the mean antero-posterior diameter of the cisterna magna by 2DUS was 4.83 mm, lower than that obtained in our study (5.60 mm). The test to prove that the antero-posterior diameter of the cisterna magna increased from 18 to 24 weeks is important because the fixed value of 10 mm for the indication of normality cannot be real in more advanced gestational ages. We believe that this low correlation measurement of the cisterna magna by 3DUS and gestational age is due to a greater difficulty in identifying the edges of the structure as it progresses toward the occipital bone. However, further studies with more cases can prove this result.

In general, the mean diameters of the transverse and anterior-posterior cerebellum and cisterna magna by 2DUS were lower than those obtained by 3DUS. In studies evaluating the volume of fetal cerebellum by 3DUS, there was also an increase in this parameter with the gestational age, both by multiplanar and virtual organ computer-aided analysis (VOCAL) methods [18–20]. There are no studies evaluating the volume of the fetal cisterna magna by 3DUS throughout gestation. Other studies using other softwares such as three-dimensional volume contrast imaging C-plane have shown a positive correlation between the biometrics of the vermis and gestational age [8–21].

We observed in this study a statistically significant difference in the measurement of transverse and anterior-posterior cerebellum and cisterna magna by 2D and 3DUS using the 3D XI (multislice view) software. The program multislice view (Samsung-Medison, Seoul, Korea) allows the evaluation of multiple plans of sequential and adjacent images arranged on the screen, being the thickness of the slices determined by the operator. In this study, we evaluated an average of 25 plans with 0.5 mm thickness between them, from the plan of the transverse cerebellar diameter measure to a plan in which the usual outline was lost and the bone of the skull base could be observed. We used as the final value the greatest measure found, unlike the 2DUS in which we used a single measure. We believe that the measures taken by 3DUS should be more reliable because it corrects any small displacement of the sound beam transducer, which can compromise the 2D measure. However, the biggest inconvenience of this technique, which makes its application in clinical practice difficult, is the long time required to perform the measurements, an average 180 seconds.

We noticed in this study an ICC value > 0.66 for all length measurements of the fetal cerebellum and cisterna magna through 3DUS, except for the intraobserver measure of the cisterna magna (ICC = 0.287). Such a result could assume a low reproducibility of the new method; however, by Bland-Altman plot it was observed that the intraobserver mean difference was similar to the cerebellum and cisterna magna (0.16 and −0.13 mm, resp.). Thus, both measurements show visually good reproducibility in the graphics. We believe that the low value of ICC is due to the small number of cases we evaluated, and as this is a laborious technique with average rating of 25 plans per volume, with a mean time of 180 seconds, it may have contributed to this low value of ICC. However, the real applicability of the method can only be testified in further studies evaluating pathological cases of fetal central nervous system.

We believe that the great importance of this study is the evaluation of borderline cases, such as an anterior-posterior diameter of the fetal cisterna magna of 9.0 mm and a transverse cerebellar diameter of 16 mm at 18 weeks of gestational age. In these cases the 3DUS with the program multislice view can help in decision making between normal and pathological cases, modifying the prenatal, and counseling of the parents.

In summary, this was the first study that compared the measurements of the transverse and antero-posterior fetal cerebellum and cisterna magna diameters by 2D- and 3DUS using the 3D XI (multislice view) software. The measurements the length of the fetal cerebellum and cisterna magna by 3DUS were significantly higher than those obtained by 2DUS. The length measurement of the cisterna magna by 3DUS showed low correlation with gestational age. Measures the length of the cerebellum and cisterna magna by 3DUS proved to be reproducible.

## Figures and Tables

**Table 1:** Comparison and difference between the diameter (mm) measures of the fetal transverse cerebellar (mm) obtained by two- and three-dimensional ultrasonography.

Cerebellum	*n*	Min	Max	Mean	SD	10th	25th	50th	75th	90th
2DUS	69	18.14	29.10	22.33	3.01	18.59	19.42	21.75	24.82	26.81
3DUS	69	18.06	29.34	23.09	3.16	19.42	20.13	23.00	26.07	27.17
3DUS-2DUS	69	−0.34	2.31	0.76	0.57	0.08	0.34	0.68	1.09	1.52

2DUS: two-dimensional ultrasonography; 3DUS: three-dimensional ultrasonography; SD: standard deviation.

**Table 2:** Comparison of different measures and the antero-posterior diameter of the fetal cisterna magna (mm) obtained by two- and three-dimensional ultrasound.

Cisterna magna	*n *	Min	Max	Mean	SD	10th	25th	50th	75th	90th
2DUS	69	3.18	9.32	5.60	1.33	4.01	4.70	5.43	6.44	7.28
3DUS	69	4.19	10.95	6.62	1.41	4.99	5.61	6.36	7.77	8.43
3DUS-2DUS	69	−1.27	3.84	1.02	0.83	0.00	0.52	1.05	1.53	1.93

2DUS: two-dimensional ultrasonography; 3DUS: three-dimensional ultrasonography; SD: standard deviation; *n*: number of case in each gestational age.

**Table 3:** Measurements of the percentiles 5, 10, 50, 90, and 95 of fetal transverse cerebellar diameter (mm) obtained by three-dimensional ultrasonography.

	GA	*n *	Mean	SD	Min	Max	5th	10th	50th	90th	95th
Cerebellum 3DUS	18	10	19.30	0.82	18.06	20.91	18.26	18.46	19.47	19.88	20.39
	19	10	19.99	0.78	18.82	21.47	18.89	18.96	20.03	20.73	21.10
	20	8	20.89	0.92	19.73	22.30	19.83	19.93	20.72	21.92	22.11
	21	10	22.95	1.67	20.84	26.76	21.26	21.68	22.64	24.53	25.64
	22	11	24.32	1.33	22.41	26.87	22.86	21.68	23.05	24.53	25.64
	23	10	25.73	1.15	23.51	26.98	23.90	24.29	26.07	26.78	26.88
	24	10	27.92	0.89	26.83	29.34	26.91	26.99	27.85	28.99	29.16

	Total	69	23.09	3.16	18.06	29.34	18.80	19.49	23.00	27.15	28.16

GA: gestational age; *n*: number of patients in each gestational age; SD: standard deviation; 3DUS: three-dimensional ultrasonography.

**Table 4:** Measurements of the percentiles 5, 10, 50, 90, and 95 of the antero-posterior diameter of the fetal cisterna magna (mm) obtained by three-dimensional ultrasonography.

	GA	*n *	Mean	SD	Min	Max	5th	10th	50th	90th	95th
Cisterna magna 3DUS	18	10	5.72	0.58	4.98	6.85	4.98	4.99	5.63	6.41	6.63
	19	10	5.47	0.81	4.22	6.60	4.32	4.43	5.45	6.29	6.44
	20	8	5.95	0.68	4.89	7.09	5.06	5.23	6.01	6.67	6.88
	21	10	7.62	1.50	4.19	9.20	4.96	5.74	7.95	9.01	9.11
	22	11	7.11	1.80	4.40	10.95	4.70	4.91	7.83	9.01	9.11
	23	10	7.34	1.22	5.22	9.38	5.69	6.16	7.39	8.67	9.02
	24	10	6.97	1.21	5.03	8.71	5.32	5.62	7.00	8.37	8.54

	Total	69	6.62	1.41	4.19	10.95	4.63	4.99	6.36	8.35	8.88

GA: gestational age; *n*: number of patients in each gestational age; SD: standard deviation; 3DUS: three-dimensional ultrasonography.
